# The change of procalcitonin concentrations after tracheostomy at bedside

**DOI:** 10.1186/2197-425X-3-S1-A240

**Published:** 2015-10-01

**Authors:** K Sato, M Okajima, T Noda, Y Koshida, T Taniguchi

**Affiliations:** Intensive Care Unit, Kanazawa University Hospital, Kanazawa, Japan

## Intr

Serial Procalcitonin (PCT) measurement has become a part of routine Intensive care unite (ICU) practice to monitor infections. Tracheostomy is one of the most common surgical procedures in ICU. In addition to procedure itself, complications of tracheostomy such as hemorrhage, airway complications, and pneumothorax may have an influence on daily PCT concentrations.

## Objectives

To investigate the relationship between the change of PCT and complications of tracheostomy in ICU.

## Methods

We conducted a retrospective chart review study of adult ICU patients at Kanazawa university hospital from January 2010 to January 2015. We included adult patients (>18 year-old) performed tracheostomy at the bedside and measured daily PCT as a marker of infection and collected results of PCT both within 12 hour before and 12-24 hour after tracheostomy. Alert PCT was defined as a PCT ≥0.5 ng/mL that was not decreasing from previous level. Perioperative complications including new onset of coincidental bacterial infections were investigated. We also collected the changes of markers including C-reactive protein, white blood cell count, SIRS, and SOFA score.

## Results

A total of 102 patients were enrolled. The mean time to tracheostomy was 8.4 ± 6.4 days. A total of 6 perioperative complications: hemorrhage (n = 1), airway complications (n = 2), pneumothorax (n = 2), bronchospasm (n = 1), and 2 coincident bacterial infections: blood stream infection (n = 1), catheter related blood stream infection (n = 1) were reported. Tracheostomy related death was not observed. Only 10% of all patients showed alert PCT. Multivariate stepwise logistic regression analyses adjusting time to tracheostomy, SOFA score, and steroid usage revealed that perioperative complication was independently associated with alert PCT. Receiver operating characteristic curves (ROC) were generated to evaluate the predictive ability of the change of PCT (ΔPCT = PCT before tracheostomy - PCT 12-24 hour after tracheostomy) for perioperative complications and showed ΔPCT was a most reliable marker with area under the ROC curve of 0.99. The optimal cut off point of ΔPCT was −0.04 ng/mL with 100% of sensitivity and 97% of specificity.

## Conclusions

The present study strongly suggests that PCT is a reliable marker to monitor the patients' course after tracheostomy in ICU.

